# Scrap tyre recycling process with molten zinc as direct heat transfer and solids separation fluid: A new reactor concept

**DOI:** 10.1016/j.mex.2016.05.003

**Published:** 2016-05-09

**Authors:** Frank Riedewald, Kieran Goode, Aidan Sexton, Maria J. Sousa-Gallagher

**Affiliations:** a Composite Recycling Ltd, The Rubicon Centre, CIT Campus, Bishopstown, T12 Y275, Cork, Ireland; b Sexton Kennedy Consulting Ltd., Cork, Ireland; c Process & Chemical Engineering, School of Engineering, University College, Cork, Ireland

**Keywords:** Tyre pyrolysis by direct heat contact with molten zinc and in-situ sink-float separation of recovered carbon black (rCB) and steel, Tyre pyrolysis, Molten metal, Direct heat transfer, Sink-float separation

## Abstract

Every year about 1.5 billion tyres are discarded worldwide representing a large amount of solid waste, but also a largely untapped source of raw materials. The objective of the method was to prove the concept of a novel scrap tyre recycling process which uses molten zinc as the direct heat transfer fluid and, simultaneously, uses this media to separate the solids products (i.e. steel and rCB) in a sink-float separation at an operating temperature of 450–470 °C.

This methodology involved:

• construction of the laboratory scale batch reactor,
• separation of floating rCB from the zinc,
• recovery of the steel from the bottom of the reactor following pyrolysis

## Graphical abstract

**Figure:**
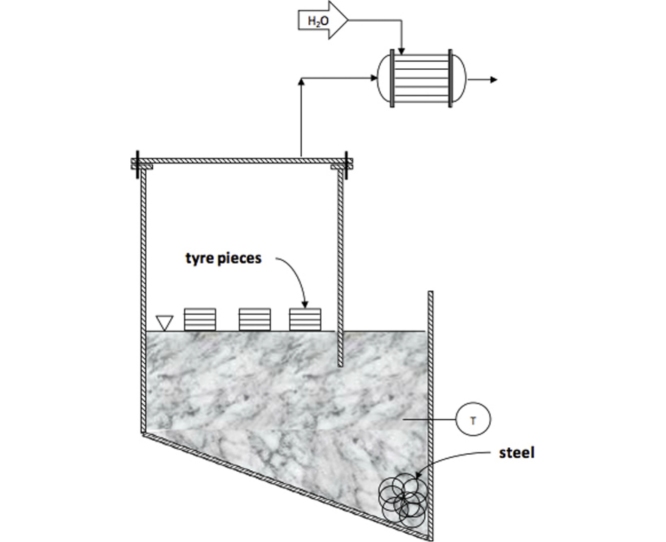

## Method details

Experiments on a laboratory scale version of a proposed tyre pyrolysis process [1] were conducted to demonstrate the feasibility of separation of the solid tyre pyrolysis products (i.e. steel and recovered carbon black (rCB)). The proposed tyre pyrolysis process uses molten zinc as the direct heat transfer and separation media for the solid pyrolysis products (i.e. steel, which sinks, and rCB, which floats on the molten zinc). The essential features of the pyrolysis reactor [1], [2], namely; its U-shaped structure, one leg closed to the atmosphere (the pyrolysis chamber), and the other leg open to the atmosphere (metal recovery chamber) with the sloped bottom were duplicated for the laboratory scale version.

## Description of the laboratory process

**Fig. 1:**
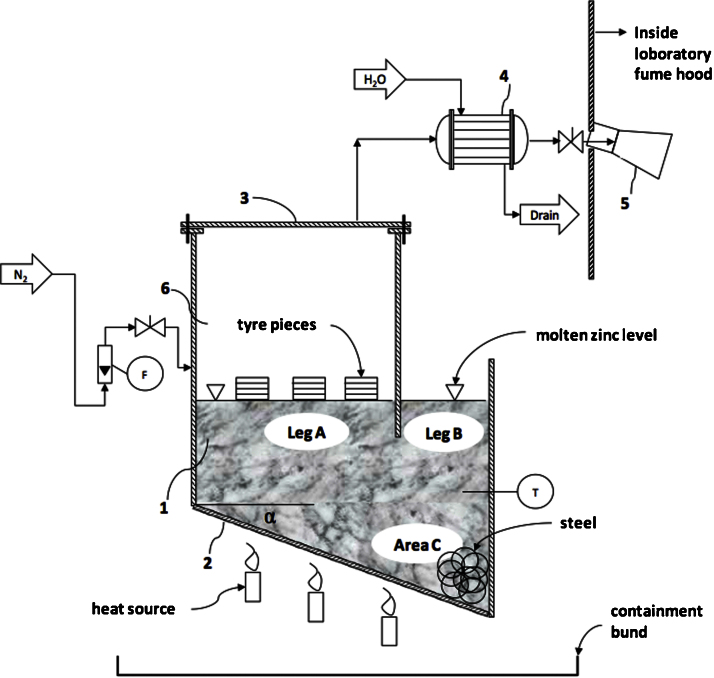
Layout and P&ID of the laboratory scale tyre pyrolysis plant. (T = temperature gauge, F = N_2_ flow meter (rotameter), N_2_ = nitrogen, α = 45°; numbers see text).

**Fig. 2:**
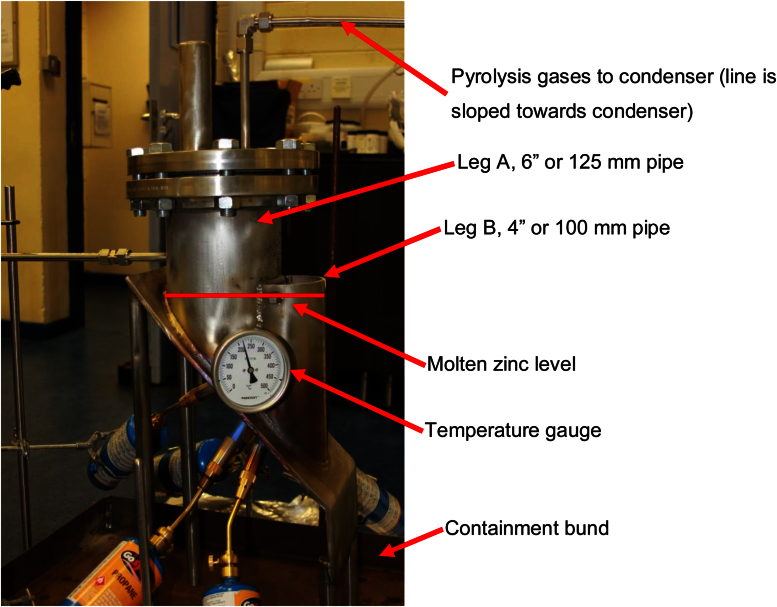
Side view of the reactor (photo taken during the heat-up phase).

**Fig. 3:**
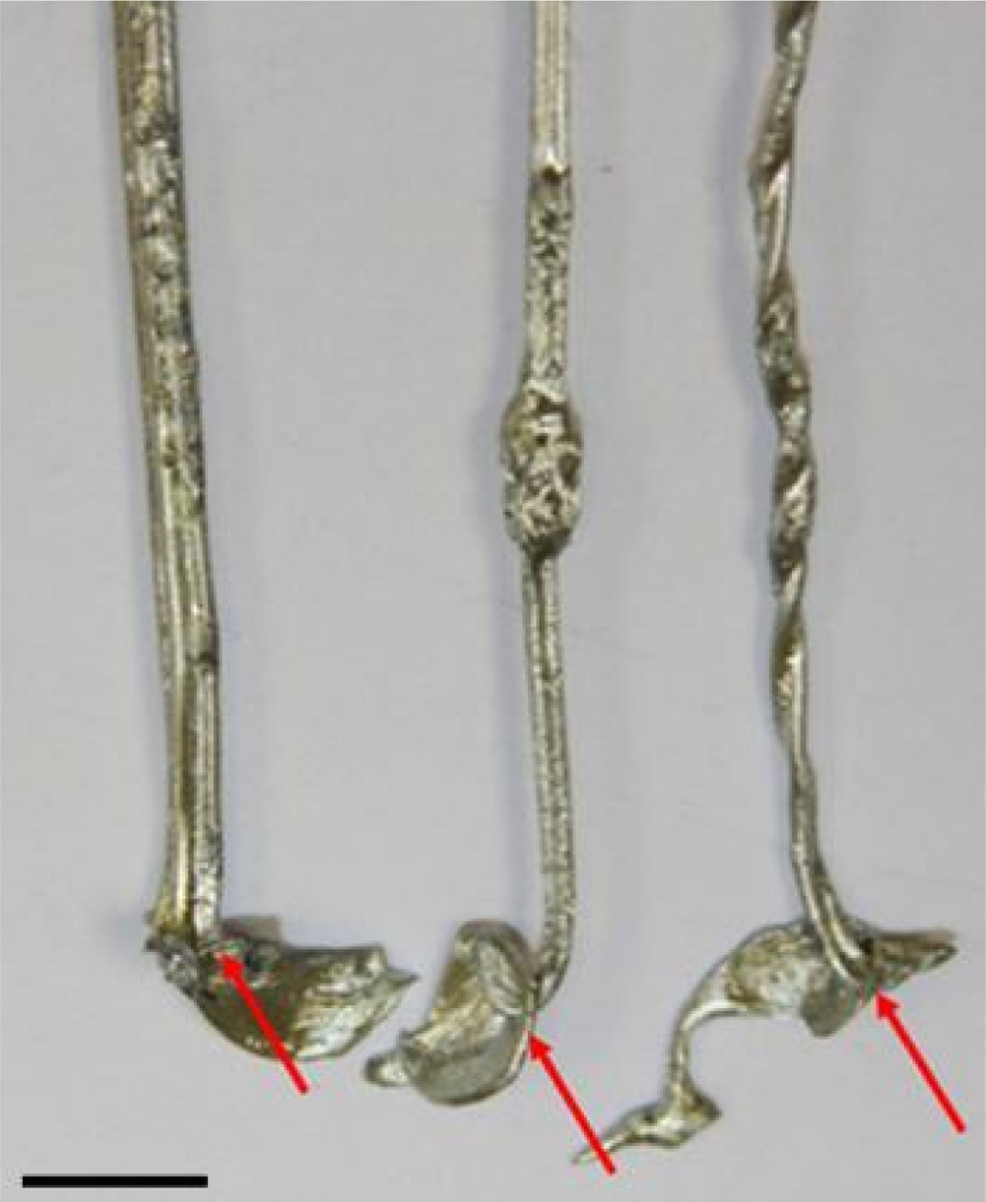
Steel wires from tyres (arrow) recovered with a steel wire hook from the bottom of Leg B (bar = 10 mm).

**Fig. 4:**
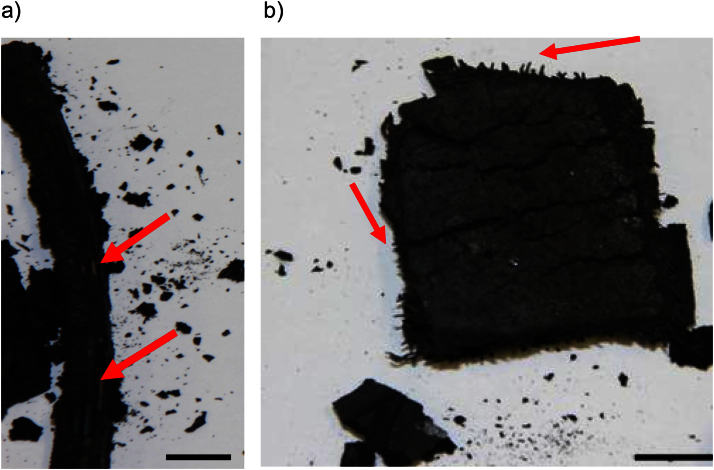
Samples of the pyrolysed tyre fragments recovered from Leg A. a) from the rim, b) from the tread. The arrows point to steel wires still embedded in the pyrolysed tyre pieces (bar = 10 mm).

A P&ID of the laboratory scale tyre recycling process is presented in Fig. 1. Fig. 2 provides an image of the reactor in the laboratory. The pyrolysis vessel (Fig. 1; 1) was manufactured from a 6” (125 mm) and a 4” (100 mm) ANSI schedule 40, 316L stainless steel pipe (6” pipe = 7.1 mm wall thickness, 4” pipe = 6.02 mm wall thickness). A 7 mm thick 316L stainless steel plate was welded onto these pipes as the bottom wall (Fig. 1; 2) of the pyrolysis vessel which was sloped 45°. Both legs of the pyrolysis vessel can be opened; leg A by unbolting the top flange (Fig. 1; 3) which ensured an airtight chamber in leg A (once the molten zinc remains above the lower edge of the separation plate between the two legs). Leg B was always open to the atmosphere allowing continuous access to the molten zinc. The pyrolysis gases generated by the tyre pyrolysis process were condensed by a water-cooled heat exchanger (Fig. 1; 4) and collected in a 100 mL laboratory glass beaker (Fig. 1; 5) inside a fumehood. The formation of an explosive atmosphere within the pyrolysis vessel (Fig. 1; 6) and downstream equipment during the experiments was prevented by continuously sweeping the pyrolysis vessel with nitrogen ensuring the safety of the experiment [3]. Since the pyrolysis chamber (Leg A) was continuously vented through the condenser, no pressure buildup occurred in the chamber.

## Molten zinc

Zinc granulate (2.2 L or 14.3 kg) was used to fill the pyrolysis reactor to the required molten zinc level above the lower edge of the separation plate between Leg A and Leg B, as indicated in Fig. 1. The relevant physical properties of molten zinc at the operating temperature of 450 °C are given in Table 1. The zinc was reagent pure, supplied as zinc metal granular and was not further purified.

## Tyre sample

A scrap car tyre, Firestone Multihawk 165/65R14 79T, was obtained from a tyre retailer. From the tyre tread (area in contact with the road) and the tyre bead bundle (part of the tyre in contact with the wheel rim), rubber pieces with a maximum size of 50 mm square and 100 mm × 40 mm were cut. These locations were chosen as they contain steel wires.

## Nitrogen

The nitrogen gas used in the experiments was certified 99.999% pure.

## Operational procedures

All experiments were carried out with molten zinc at an operating temperature of 450–470 °C, with the pyrolysis reactor (leg A) sealed and under a nitrogen atmosphere. The safety aspects of executing such an experiment must not be underestimated, as the experiment involves molten zinc at high temperatures. Details on the safety measures implemented such as two-man operation, special personnel protection equipment, nitrogen sweeping of the chamber and others were reported previously [3].

The tyre pieces were placed side by side in leg A. Leg A was closed and nitrogen sweep inerted at a flowrate of 10 Nm^3^/h for one minute to purge oxygen from the pyrolysis chamber before the heat was applied to prevent oxidation of the tyre rubber. Thereafter, the nitrogen flow was reduced to about 1 Nm^3^/h to maintain an inert atmosphere within the pyrolysis chamber at all times.

The operating temperature of the molten zinc was measured using an Ashcroft temperature gauge (range of 0–500 °C, with a 1% ASME B40.3, Grade A accuracy) inserted into a thermowell manufactured from 316L stainless steel. The operating temperature was controlled by manually adjusting up to seven blow torches.

Once achieved, the operating temperature of 450–470 °C was maintained until pyrolysis gases could no longer be visually detected entering the glass flask (Fig. 1; 5) located inside a fume hood, which extracted the non-condensable gases to the atmosphere. On reaching this experimental endpoint, the burners were turned off and the chamber was allowed to cool naturally to ambient temperature. Once the reactor cooled to ambient temperatures the nitrogen was stopped. Finally, leg A of the pyrolysis reactor was opened for inspection.

Tyre steel wires, which migrated under gravitational force to the bottom of leg B (i.e., Area C (Fig. 1)), were recovered or “fished out” from the bottom of leg B at the operating temperature of 450–470 °C by two twisted steel wires each 300 mm long. The end of one of these two wires was bent into a small hook, which facilitated the recovery of the steel wires, which sank and migrated to the bottom of leg B due to their higher density with respect to the molten zinc (see Table 1), as shown in Fig. 3.

Some of the tyre pieces cut from the tread of the tyre did not disintegrate during the experiment, and it was possible to remove them intact from the reactor as shown in Fig. 4 (b). Comparing the weight of these pieces to their original weight showed a weight loss of more than 50%, which indicates that most, if not all, of the rubber was pyrolysed, since new tyres are typically 40–60% rubber, as indicated in Table 2. A Sartorius ED4202S laboratory scale (max 4200 g; d = 0.01 g) was used to weigh the tyre pieces.

## Sink-float separation of recovered carbon black and steel

Due to its higher density (see Table 1) steel sinks in molten zinc. The rCB floats on the molten zinc, because rCB containing more than 96% carbon is not miscible with molten zinc at the operating temperatures [4] and its density is lower than that of molten zinc. This sink-float separation of the steel and rCB was confirmed experimentally by the laboratory scale experiments and, hence, achieving the aim of the experiments.

Some of the steel tyre wires recovered from the bottom of leg B i.e. Area C (Fig. 1) are shown in Fig. 3. As is evident from Fig. 3 the recovered steel wires are coated with zinc. Two possibilities were identified to deal with this zinc drag out. The recovered steel could be sold off coated with the zinc, or the recovered steel could be placed in an oven [5], in which the zinc would be melted off and recycled back to the pyrolysis vessel.

During the laboratory scale experiments, not all the wires sank into the melt, as some were still embedded in the pyrolysed tyre pieces as can be seen from Fig. 4. Clearly, for the wires to sink, they must be separated from the rCB and be in contact with the molten zinc. Hence for the rCB/steel separation to work in the full scale plant, the rCB must be removable from the steel wires within the pyrolysis reactor.

Two pyrolysed tyre pieces recovered from the laboratory scale experiments are shown in Fig. 4. These pieces were so brittle that it was difficult to recover them from leg A without breaking them as the rubber holding the carbon black, steel and other components, as indicated in Table 2, together was no longer present. The piece shown in Fig. 4 (a) is from the rim of the tyre containing relatively few and large diameter steel wires. The rCB broke off easily, when the tyre piece was handled during removal from leg A. The piece shown in Fig. 4 (b), on the other hand, was from the tread of the tyre and contains a steel matrix comprising of small diameter wires providing better structural stability to the tyre piece now being composed of rCB and steel only. However, the rCB may easily be separated from the wires by applying a small amount of force as the rCB was very brittle. Nevertheless demonstration scale test (see Table 3) must be performed to demonstrate that the rCB can indeed be separated from the steel in-situ as proposed [1]. However the laboratory scale experiments successfully demonstrated the separation of the rCB (δ = 1800–2100 kg/m^3^) and steel (δ = 7400-8000 kg/m^3^), can be facilitated by molten zinc (δ = 6508 kg/m^3^) in a sink-float separation.

## Additional information

Every year about 1.5 billion tyres are discarded worldwide creating a large amount of solid waste, but also a largely untapped source of raw materials [6]. Recycling of tyres is difficult because they are designed to be durable and tough since they have to withstand enormous forces during their working life [7]. This toughness and durability is achieved by combining the properties of materials such as rubber, steel, fabrics and carbon black and other components in a composite [8], [9]. The average tyre composition is given in Table 2.

Scraped tyres are used in various ways:

(1) Granulated tyres or rubber crumb is used for running tracks and children’s playgrounds,
(2) Whole tyres are used in civil engineering projects such as bank stabilisation on motorways [6].
(3) However, in some countries up to 60% of the collected scrap tyres are used as fuel in cement kilns recovering the calorific value only [10].

Pyrolysis is a promising technology capable of recycling tyres in a self-sustaining manner by utilising the heat content of the non-condensable gases [9]. Pyrolysis involves the use of heat in the absence of oxygen breaking down the organic tyre compounds (rubber, nylon) into products such as pyrolysis oil and gases, but also having the potential to recycle the solid products (rCB and steel).

Current tyre pyrolysis processes depend on technologies such as rotary kilns, entrained beds, screw kilns or batch reactors [8], [9], [11], which, despite many attempts, have commercially not been successful [12]. The process described here using molten zinc as a direct heat transfer and separation media provides an alternative to the current practice of using rotary kilns or indirect heat transfer. The laboratory scale experiments described herein are only the first step towards a full scale process. With each scale-up other issues can be addressed, such as whole tyre kinetics, yields and product quality as outlined in Table 3.

The yield and quality of the various products of scrap tyre, such as pyrolysis oil, rCB and steel, using conventional pyrolysis have been reported [8], [13], [14]. Typical pyrolysis products of tyres are on average of ∼55 wt% pyrolysis oil, ∼30 wt% rCB, ∼10 wt% steel and ∼5 wt% non-condensable gas [11]. This gas may be burned to self-sustain the tyre recycling process [8], [9], [11], [15]. But, as the composition and yields are also dependent on the process conditions [8], [9], [11], the yields and product composition from this molten zinc process may be different to the results reported from other processes. Actual data of the yields, kinetics and other pertinent information can only be obtained from future experiments as outlined in Table 3. Experiments on the pilot plant scale showed that a whole tyre can be pyrolysed within 20 min [16], which is significantly faster than conventional processes which have processing times of 2–4 h [8], [9], [11]. Moreover the analysis of the pyrolysis oil obtained from the pilot plant indicates that the oil from the molten zinc process is virtually identical to the oil from other processes, except that it contains a higher d-limonene concentration as the processing time is lower minimising cracking of the d-limonene [16].

Molten salt instead of molten zinc may be used to pyrolyse tyres [17]. A major drawback of salt over zinc is that the rCB will be contaminated with salt [18] and, as a result, another unit operation to remove the salt from the rCB would be required. The zinc based process discussed in this paper has, therefore, a significant advantage over the salt based process.

## Figures and Tables

**Table 1:** Physical properties of molten zinc, carbon black and steel [19], [20] in comparison to water [20].

Compound	Density (kg/m^3^)	Surface tension (N/m)	Viscosity (Pa·s)	Melting point (°C)	Vapour pressure (Pa)
Zinc (molten)	6508 (at 476 °C)	0.755 at 450 °C	0.003254 at 476 °C	419	100 (at 477 °C)
Carbon black	1800–2100	–	–	3550	–
Steel	7400–8000	–	–	>1300	–
Water (25 °C)	1000	0.072	0.001003	0	3000

**Table 2:** Average composition of new tyres [7].

Component	Wt%
Rubber (natural or synthetic)	40–60
Carbon black	27–30
Steel (wires)	14–16
Fabric, fillers, accelerators	16–17
Zinc oxide	1.9
Sulphur	1.1

**Table 3:** Development stages of the tyre recycling process from laboratory to demonstration scale.

Stage of development	Molten zinc (kg)	Process conditions	Results attainable
Laboratory scale	2.2	Tyre pieces; Batch	Proof of concept of separation: rCB/steel and vapour i.e. P-oil
Pilot scale	900	Whole tyres; Batch	Composition of P-oil; Kinetics of whole tyre pyrolysis; Separation steel/rCB of whole tyre in batch mode
Demonstration scale	∼12,000	Whole tyres; Continuous	Continuous separation steel/rCB; Composition of rCB incl. zinc conc.; Yields (P-oil, steel, char); Quality of recovered steel; Losses i.e. zinc; Composition of non-condensable gas; Effects, if any, of metallurgy on kettle life time and product separation/efficiency and product quality.
